# Knee function, quality of life, pain, and living conditions after distal femoral resection knee arthroplasty for non-tumor indications

**DOI:** 10.1186/s12891-022-06104-z

**Published:** 2023-01-06

**Authors:** Yasemin Corap, Michael Brix, Julie R. Brandt, Claus Emmeluth, Martin Lindberg-Larsen

**Affiliations:** Orthopaedic Research Unit, Department of Orthopaedic Surgery and Traumatology, Odense University Hospital, Department of Clinical Research, University of Southern Denmark, Odense, Denmark

**Keywords:** Resection knee arthroplasty, Quality of life, Knee function, Oxford knee score, Copenhagen knee rom, EQ-5D

## Abstract

**Background:**

Distal femoral resection knee arthroplasty is a limb salvage procedure. The impact of distal femoral resection arthroplasty on patient function and health status is unknown. The aim of this study was to report knee function, quality of life, knee pain, and living conditions after distal femoral resection knee arthroplasty for non-tumor indications.

**Methods:**

Of 52 patients (52 knees) undergoing distal femoral resection knee arthroplasty in a single institution between 2012 and 2021, 22 were excluded as 3 patients had ≤90 days follow-up, 6 had died, and 13 declined or were unable to participate for unrelated reasons. Thus, 30 patients were included and interviewed by telephone in March 2021 (mean follow-up 3.5 years after surgery). Patient completed the Oxford Knee Score (0–48, 48 best), EQ-5D-5L, and the Copenhagen Knee ROM, and information on pain and living conditions was obtained.

**Results:**

The mean age was 67.9 years (SD 13.6), and 21 (70%) were female.

Mean total Oxford Knee Score was 29.9 (SD 10.5), mean Copenhagen Knee ROM flexion was 116° (SD 21.6), and mean extension was − 2° (SD10.1). Mobility aids were used by 18 (60%) patients, i.e. a cane (30%), walker (26.7%) or wheelchair (3.3%).

Mean EQ-5D_index_ score was 0.70 (SD 0.22) and mean EQ-5D VAS score was 55.4 (SD 23.9). Nine (30%) patients used paracetamol or NSAID and 2 (6.7%) used opioids for knee pain. Mean VAS knee pain score was 1.30 (SD 2.2) at rest and 2.8 (SD 3.1) when walking.

Most (90%) patients lived in their own home, with only 3 patients in nursing homes. Two-thirds (66.7%) required no home care, 5 (16.6%) received home care 1–2 times over 2 weeks, and 5 (16.6%) every day.

**Conclusion:**

Distal femoral resection knee arthroplasty appears to be a viable treatment option for non-tumor indications. Acceptable patient outcomes were achieved in terms of functional status and quality of life, especially considering treatment alternatives such as femoral amputation.

## Introduction

Knee arthroplasties appear to be increasing in frequency [1], and hence the need for revision knee arthroplasties is also expected to increase due to periprosthetic fractures or severe bone loss. Treatment options after failed total knee arthroplasty are limited, and distal femoral resection knee arthroplasty, which is a limb salvage procedure, may be needed more frequently in the future [2].

Distal femoral resection knee arthroplasty may be a viable option for several non-oncologic indications and has additional therapeutic opportunities as long extremity bone reconstruction [3–5], but the patients requiring this type of surgery are often elderly and with high medical comorbidity. Only limited data exist on the outcome of distal femoral resection knee arthroplasty for non-oncologic indications, but most of the recently published case series suggest it may be a reasonable treatment option [6–11]. The procedure appears to be a relatively safe option in terms of surgical complications and mortality [12].

Although the patient’s functional status and quality of life influence the choice of treatment, few studies have addressed function, pain, and quality of life after surgery with distal femoral resection knee arthroplasty.

The aim of this study was to report patient-reported knee function, quality of life, knee pain, and living conditions after distal femoral resection knee arthroplasty for non-tumor indications.

## Matrial and methods

### Study design

This was a cohort study of patients treated with distal femoral resection knee arthroplasty in a single center between January 2012 and December 2021. The study cohort was identified retrospectively, but all patients were invited to participate in a supplementary clinical and questionnaire follow-up in 2021 as part of the current study. Mean follow-up was 3.5 years (range 124 days to 9 years). Patient safety results for the entire cohort has been reported in a previous publication [12].

### Patients

**Fig. 1:**
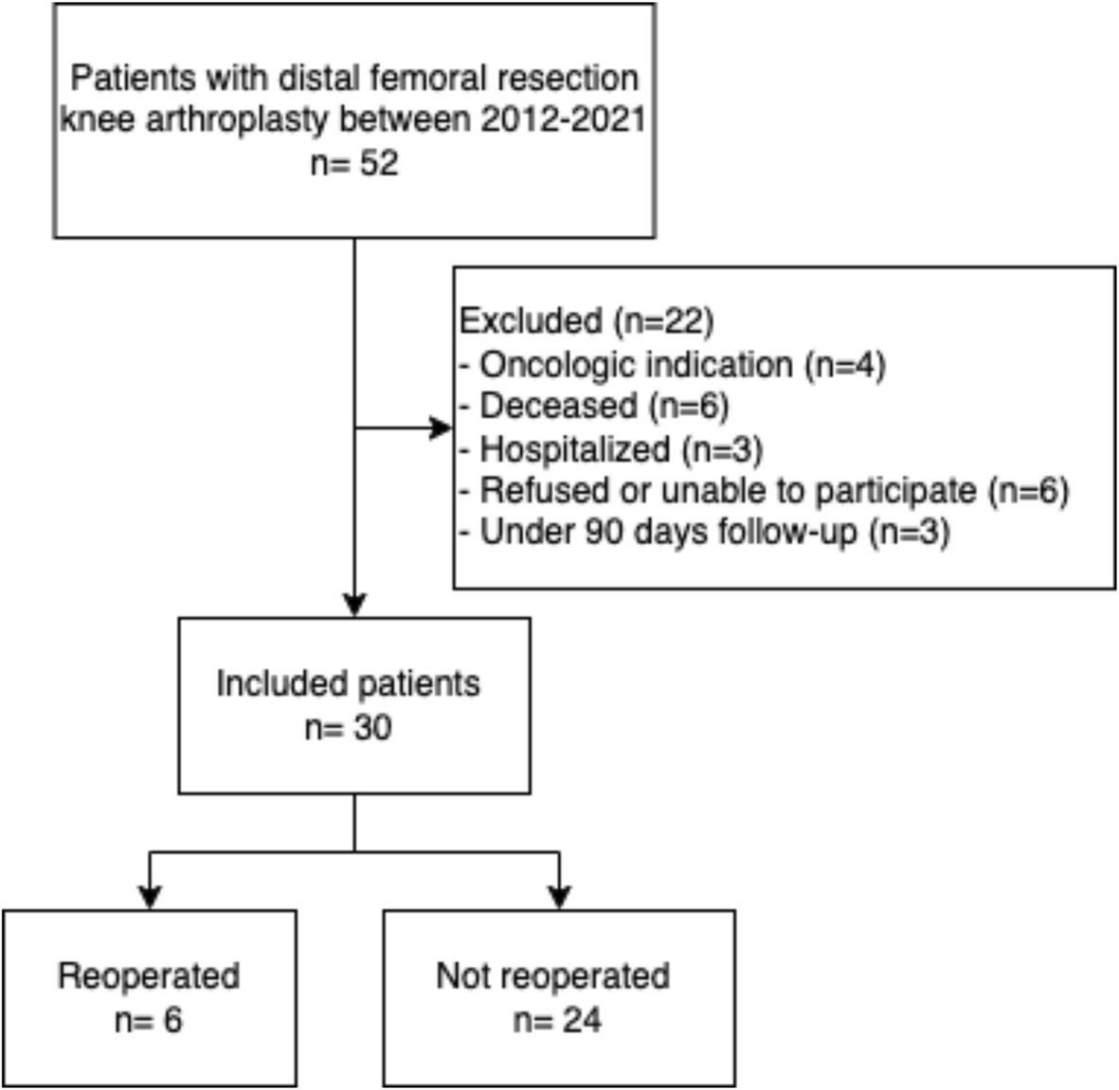
Flowchart of patient inclusion in the study

During the study period, 52 distal femoral resection knee arthroplasties were performed. Of these, 22 were excluded from further analysis as 3 patients had ≤90 days follow-up, 6 had died, 6 declined participation, 3 were unable to participate due to hospitalization unrelated to the knee surgery, and 4 had an oncologic indication for the arthroplasty (Fig. 1). Hence, 30 patients agreed to participate in a follow-up telephone interview and were included in the study. The surgical indications were failure of osteosynthesis (*n* = 6), primary fracture treatment (*n* = 3), periprosthetic fracture (*n* = 12), and revision arthroplasty with severe bone loss (*n* = 9). Before the interview, all patients received questionnaires and written information about the interview.

Two consultant knee revision surgeons (CE and MLL) performed the procedures. The The GMRS – Global Modular Replacement System (Stryker) prosthesis was used in cases performed before 2016 (*n* = 7) and the LPS – Limp Preservation System (DePuy Synthes) was used from 2016 (*n* = 23).

All patients were administered 1 g tranexamic acid preoperatively and prophylactic antibiotic treatment with 1 g Dicloxacillin or in case of allergy 1,5 g Cerfuroxime. In non-revision cases prophylactic antibiotic treatment were administered at 8, 16 and 24 h after surgery and in revision cases (prosthesis exchange procedures) continued until analysis of intraoperative biopsies (*n* = 5) were finalized and microbiology results were confirmed as negative. No drains were used postoperatively.

Bone cement was used for fixation on the femural component. Surface baseplate cementation was used on the tibial prosthesis with cementless sleeve and stem. No silver-coated implants were used.

**Fig. 2:**
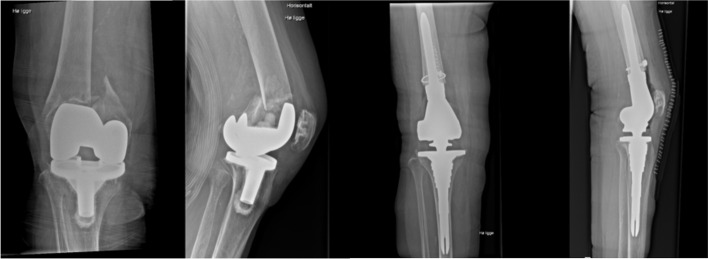
Periprosthetic Knee Fracture treated with Distal Femoral Resection Knee Arthroplasty.

Within 24 h of surgery all patients received physiotherapy to help standing up and walk using a walking aid with full weight bearing on the operated leg. An example of periprosthetish knee fracture treated with distal femoral resection knee arthroplasty can be seen in Fig. 2.

### Outcomes

#### Knee function

Patients completed the Oxford Knee Score (OKS, score range 0–48, where 48 represents optimal knee function). OKS is validated to measure function and pain after knee replacement [13]. Patients also completed the Copenhagen Knee ROM [14], where patients report their own active knee range of motion with the help of pictures.

Quality of life.

Quality of life data were obtained using the EQ-5D-5L [15], which includes five questions asking about the dimensions of mobility, self-care, usual activities, pain or discomfort, and anxiety or depression, each with five response options from No problems to Extreme problems/Unable to perform. Patients also rate their own health on a vertical 0–100 scale (EQ-5D VAS) with endpoints of best/worst health you can imagine. The responses to the five dimensions can be converted into a weighted index score ranging from 0 to 1 that reflects population health preferences, where 0 represents the state of being dead and 1 represents full health; negative scores suggest that a health state is considered worse than being dead. Scores in the Danish EQ-5D-5L value set range from − 0.757 to 1 [16].

#### Knee pain

A horizontal 10 cm visual analogue scale (VAS) was used to evaluate pain experienced in the area of the distal femoral resection knee arthroplasty. The patients were asked to score the intensity of pain experienced during rest and after taking 5 steps (resting/ambulation). The VAS scale ranged from 0 (no pain) to 10 (intolerable pain). During the interview, patients were also asked about use of analgesics for knee pain, including type, dose, and frequency.

#### Living conditions

During the interview, patients were asked about their accommodation (own home/nursing home), home care services used, and any mobility aids used (e.g. cane, walker/walking frame, wheelchair).

### Statistics

Continuous data are presented as mean (SD) or median (interquartile range, IQR) as appropriate. Categorical data are presented as n (%) with 95% confidence intervals (CI).

Data were analyzed using SPSS version 24 (2016; Armonk, NY: IBM Corp.).

## Results

A total of 30 distal femoral resection knee arthroplasties performed in 30 patients were analyzed. Mean patient age was 67.9 years (SD 13.6), and 21 (70%) were females (Table 1).

**Table 1:** Characteristics of patients undergoing distal femoral resection knee arthroplasty for non-tumor indications between 2012 and 2021

	All indications	Not reoperated	Reoperated*
N (%)	30 (100)	24 (80)	6 (20)
Mean age (years) (SD)	67.9 (13.6)	69 (12.6)	64 (17.5)
Female (%)	21 (70)	15 (62.5)	6 (100)
BMI (SD)	27.8 (5.7)	27.5 (6.0)	29.2 (4.8)
ASA (%)			
ASA Score 1	4 (13.3)	3 (12.5)	1 (16.7)
ASA score 2	10 (33.3)	7 (29.2)	3 (50.0)
ASA score 3	16 (53.3)	14 (58.3)	2 (33.3)
ASA score 4	0	0	0
Insulin-dependent diabetes (%)	0	0	0
Non-insulin dependent diabetes (%)	2 (6.7)	2 (8.3)	0
Cardiac disease (%)	8 (26.6)	7 (29.2)	1 (16.7)
Pulmonary disease (%)	5 (16.7)	5 (20.8)	0
Immunosuppression (%)	1 (3)	1 (4.2)	0

*BMI* Body mass index; *ASA* American Society of Anesthesiologists

* Causes of reoperations were infection (*n* = 3), patella dislocation (*n* = 2) and polyethylene liner dislocation (*n* = 1) and all patients underwent a minor reoperation without exchange of femoral and tibial components

The indications for femoral resection knee arthroplasty were failure of osteosynthesis, primary fracture treatment, periprosthetic fracture, and revision arthroplasty for severe bone loss.

**Fig. 3:**
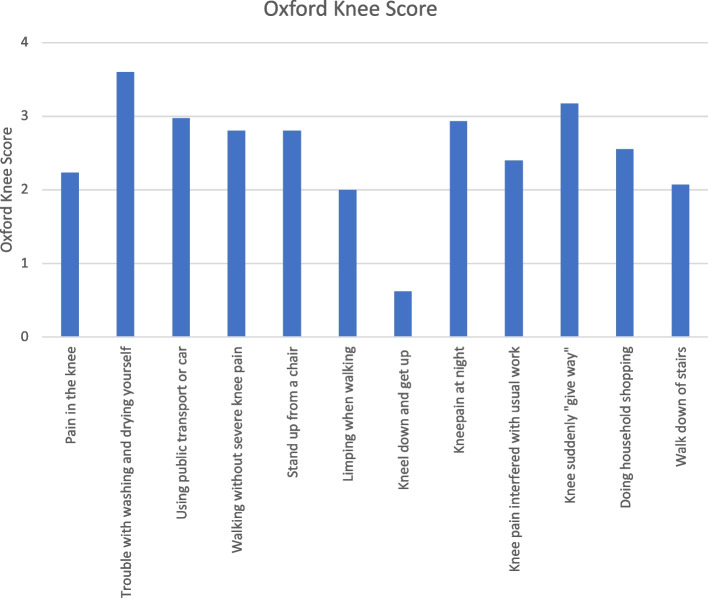
Oxford Knee Score. 0 = severe knee pain, 1=moderate, 2= mild, 3= very mild, 4=none

Mean total OKS was 29.9 (SD 10.5) (Fig. 3). Mean Copenhagen Knee ROM flexion was 116° (SD 21.6) and mean extension was − 2° (SD10.1).

Mean EQ-5D index score was 0.70 (SD 0.22), and mean EQ-5D VAS score was 55.4 (SD 23.9).

Median VAS (1–10) pain score at rest was 0 (IQR 0–2) and while walking was 2 (IQR 0–5). Nine (30%) patients used paracetamol or NSAID, and 2 (6.7%) patients used opioids for their knee pain.

**Fig. 4:**
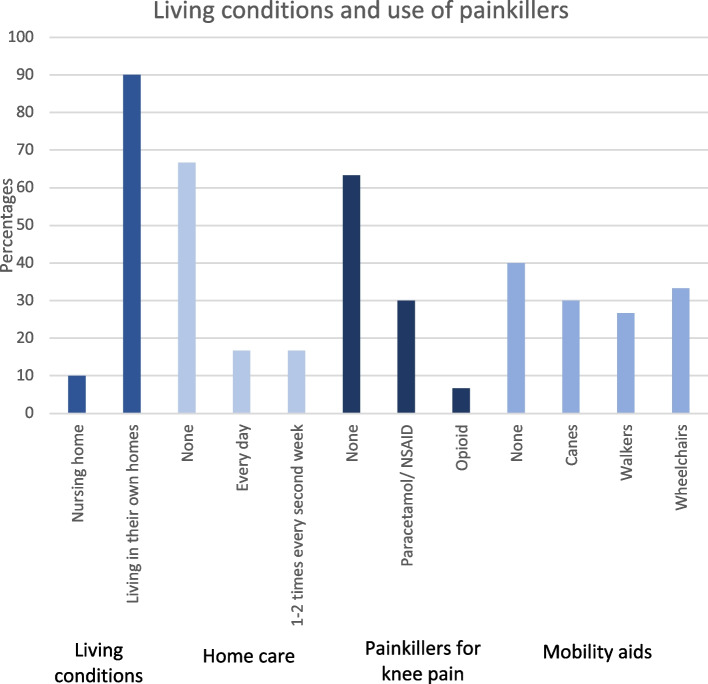
Percentages plottet against living conditions and use of painkillers after distal femoral resection knee arthroplasty

Only 3 (10%) patients were living in a nursing home at follow-up, and 27 (90%) patients were living in their own homes. Twenty (66.7%) patients did not need home care, 5 (16.7%) received home care every day, and 5 (16.7%) received home care 1–2 times over two weeks. Mobility aids were used by 18 patients (60%) in the form of canes (9), walkers (8), and wheelchairs (1) (Fig. 4.).

The six patients who underwent reoperation were analyzed separately (Tables 1 and 2), but they did not appear to have significantly worse outcome compared to those undergoing primary resection knee arthroplasty.

**Table 2:** Outcomes for patients undergoing distal femoral resection knee arthroplasty

	All indications	No reoperation	After reoperation
N (%)	30 (100)	24	6 (100)
Median VAS (IQR)			
Resting	1.3 (2.2)	1(2.3)	1 (2.0)
Ambulation	2.8 (3.1)	3 (3.3)	3 (2.7)
OKS (SD)	29.9 (10.5)	29.5 (11.1)**	31.8 (7.9)
EQ-5D index (SD)	0.7 (0.2)	0.7 (0.2)	0.7 (0.1)
EQ-5D VAS (SD)	55.4 (23.9)	57 (23.8)	49 (25.4)
CKR (SD)			
Flexion	116 (21.6)	118 (21.8)*	115 (15.5)
Extension	−2 (10.1)	−2 (10.4)*	0 (9.49)

* Missing data on 1 person

** Missing data on 2 persons

*VAS* Visual analogue scale; *OKS* Oxford knee score; *CKR* Copenhagen knee ROM

## Discussion

This study found that distal femoral resection knee arthroplasty was associated with acceptable patient function and health status at follow-up (mean 3.5 years) as patients reported mean total Oxford Knee Score of 30 points (on a 0–48 scale), wide range of motion of the knee, mean EQ-5D-5L index score of 0.70 (where a score of 1.0 represents full health), and a low degree of knee pain at rest and during walking.

The mean postoperative OKS of 30 in our study was similar to 27 reported by Girgis et al. [17] and 32 reported by Vitiello et al. [18], and higher than 19 reported by Toepfer et al. [19] after distal femoral resection knee arthroplasty. Yap et al. 2021 [20] reported OKS of 34 at one year after primary total knee arthroplasty (TKA), while a Patient Acceptable Symptoms State (PASS) value of 30 OKS points and a Treatment Failure value of 27 OKS points at 24 months after primary TKA has been reported [21]. In this perspective, an OKS score of 30 points seems to be acceptable after distal femoral resection knee arthroplasty when taking the more extensive and often acute surgery into consideration.

The mean knee flexion at follow-up in our study was 116°, which is higher than the 100° reported by Girgis et al. [17] after resection knee arthroplasty. At least 110° knee flexion is needed to be able to sit down and rise from a chair, so this value is likely to be important from the patient perspective.

The mean patient self-rated health (EQ-5D VAS) of 55 in our study was higher than the 45 reported in Girgis et al. [17]. The EQ-5D-5L index score was 0.70 in our study and can be compared to 0.51 at 24 months after transfemoral amputation in a Swedish study [22] and 0.73 at 12 months after primary TKA in a Swedish study using EQ-5D-3L [23]. These findings suggests that a better quality of life can be achieved with distal femoral resection knee arthroplasty compared to the alternative of femoral amputation, and it is encouraging that quality of life scores are no worse than those after primary total knee replacement.

Our patients reported a median VAS pain score of 2 (0–5) on ambulation. For comparison, Tandon et al. [24] reported a VAS pain score of 2 after distal femoral arthroplasty but did not distinguish between standing and ambulation. These data indicate that knee pain may not be the biggest concern after these procedures.

Nearly all (90%) of our patients were still living in their own home postoperatively, which is higher than the 57% reported by Girgis et al. [17]. Furthermore, 40% of our patients did not use mobility aids postoperatively while 30% used a cane, 27% used a walker and only 3% used a wheelchair. Girgis et al. [17] reported 21% did not need mobility aids, 7.1% used a cane, 50% used a walker, and 14.3% used a wheelchair. These differences between the cohorts might be explained by the older population in the study by Girgis et al. [17] with mean age of 82 years (range 70–94). Importantly, the data from both studies confirm that it is usually possible to retain gait function with or without a mobility aid.

Our patients who underwent resection knee arthroplasty as a reoperation achieved a mean OKS score of 28, which was only 2 points below the patients undergoing primary resection knee arthroplasty. The mean EQ-5D-5L index scorewas the same for reoperated and primary operated patients, but self-rated health (EQ-5D VAS) was lower (49) in the reoperated patients compared to 57 for the others. This could be expected, but it was encouraging that the outcome was not significantly worse after reoperation.

The limitations of our study include the study design and the relatively small number of patients, and these may limit the generalizability of our results to other settings. However, the positive results suggest that the indications for this relative rare procedure may be widened in the future as many patients could benefit from it. An important limitation is that we have no information on the deceased patients or on the other patients who did not participate in the follow-up. Hence, it is reasonable to believe that our findings overestimate the positive effect of the surgery. Furthermore, our data were collected at different times postoperatively (range 124 days to 9 years), which is not ideal as it introduces time bias, and no preoperatively data on life quality and knee function was collected. Nevertheless, our data provide evidence for what is possible to achieve regarding knee function, quality of life, knee pain, and living conditions after resection knee arthroplasty. This is important information for both surgeons and patients in the shared decision-making prior to surgery—especially as such data has previously been almost non-existent.

Other strengths of our study are the consecutive cohort and the detailed information on patient characteristics, comorbidity, knee function, and quality of life.

## Conclusion

Distal femoral resection knee arthroplasty appears to be a viable treatment option for non-tumor indications. Acceptable patient outcomes can be achieved in terms of knee function, quality of life, and living conditions, especially when compared with treatment alternatives such as femoral amputation.

## Data Availability

The data used and/or analyzed during the current study is available from the corresponding author on reasonable request.
